# The American Medical Association at Atlantic City

**Published:** 1907-08

**Authors:** 


**A Monthly Review of Medicine and Surgery.**

*EDITOR:*

WILLIAM WARREN POTTER, M. D.

All communications, whether of a literary or business nature, books for review and exchanges, should be addressed to the editor 238 DELAWARE AVE., BUFFALO, N. Y.

VOL. LXIII. AUGUST, 1907. NO. 1

### The American Medical Association at Atlantic City.

FOR the third time within seven years the JOURNAL gives editorial comment upon the meeting of this great medical organisation at the greatest seaside resort in the world. Each time the association has convened at Atlantic City its attendance has been larger and better work has been done than at any other place where it has met. Never in its history has so much good work been done as at the meeting in question.

The fifty-eighth annual session, held June 4-7, 1907, was conspicuous for several reasons. One of the most important duties of such a body is the selection of its presiding officer. President Bryant has long been famous as a parliamentarian as well as for his knowledge of professional legislative detail. His presidential address showed a complete grasp of all the questions that have any importance in the national medical councils. He dealt understandingly with the problems of medical education, of medical legislation, and of chemistry and pharmacology; and besides, as presiding officer of the house of delegates, he dispatched business with such promptitude that this medical legislative body was enabled to accomplish more useful work than at any other time since it was established.

The weekly medical journals have commented from their various viewpoints on this meeting and some of the monthly magazines have unloaded their pent up ideas and opinions, either in friendly criticism or the reverse, so that it is unnecessary for us to rake over this well plowed and harrowed ground. Our purpose is to set down nought in malice, but rather to praise the association's good deeds and to stimulate it to a continuance thereof along the present lines of progress.

We, therefore, shall limit our remarks at this time to comment upon one or two problems of great importance with which the association is now confronted. The first of these relates to medical education and is dealt with through the council on that subject headed by its chairman, Arthur Dean Bevan, of Chicago. The second problem of importance relates to medical legislation, upon which subject the association has also created a council headed by Charles A. L. Reed, of Cincinnati. The third question deals with pharmacy and chemistry, the chairman of this council being George H. Simmons.

Without disparagement to either of the other topics we unhesitatingly declare that the Council on Medical Education has in hand by far the greatest problem that has ever received the official stamp of the American medical profession. With an educated and enlightened body of medical gentlemen throughout the United States and its dependencies, there will be less need of medical legislation, while the duties of the Council on Chemistry and Pharmacy will practically be abolished. Certainly, the latter council will be forced to abandon the campaign pursued on its present lines of espionage and animadversion.

Elsewhere in this issue will be found a statement from the Council on Medical Education summarising the work of three years in an endeavor to improve the standard of the colleges in this country. The exhibit is an interesting one and should receive the attention of medical educators throughout the country. It represents a vast amount of hard work. Under the guidance of the council it is more than probable that conditions will be very much improved within a reasonable time. It is important that all state medical examining boards should be represented at the annual conferences at Chicago, and it were well if medical societies and medical schools should also send delegates.

The growth of the American Medical Association is phenomenal. The report of the general secretary shows the present membership of the association to be 27,515, an increase during the year of 3,879 members. The net income for 1906 was $325,300. The total expenses for the year were $293,385.00, leaving a net revenue of $31,915.00.

The election of officers resulted as follows: president, Herbert L. Burrell, Boston; first vice-president, Edwin Walker, Evansville, Ind; second vice-president, Hiram R. Burton, Lewes, Del.; third vice-president, George W. Crile, Cleveland; fourth vice-president, W. Blair Stewart, Atlantic City; general secretary, George H. Simmons, Chicago; treasurer, Frank Billings, Chicago; trustees, T. J. Happel, Trenton, Tenn., re-elected (1907-1910); W. W. Grant, Denver, re-elected (1907-1910); Philip Marvel, Atlantic City, re-elected (1907-1910). The other members of the board are: E. E. Montgomery, Philadelphia, 1908; A. L. Wright, Carroll, Ia., 1908; H. L. E. Johnson, Washington, D. C., 1908; M. L. Harris, Chicago, 1909; Wm. H. Welch, Baltimore, 1909; Miles F. Porter, Ft. Wayne, 1909.

The following nominations for committees were then made by the president and confirmed by the house of delegates: Committee on Medical Legislation, in place of W. L. Rodman, C. S. Bacon, Chicago. The other members of the Committee are: C. A. L. Reed, Cincinnati, chairman, 1909; Wm. H. Welch, Baltimore, 1908.

Council on Medical Education: in place of Charles H. Frasier, James W. Holland, Philadelphia. The other members of the council are: Arthur Dean Bevan, Chicago, chairman, 1909; W. T. Councilman, Boston, 1910; J. A. Witherspoon, Nashville, 1911; Victor C. Vaughn, Ann Arbor, 1908.

Committee on Transportation and Place of Session: M. L. Harris, Chicago; E. Eliot Harris, New York; W. A. Jayne, Denver; W. T. Sarles, Sparta, Wis. John C. Munro, Boston, is chairman of this committee.

Committee on Organisation: J. N. McCormack, Bowling Green, Ky.; George H. Simmons, Chicago; Philip Mills Jones, San Francisco.

Board of Public Instruction on Medical Subjects: J. G. Clark, Philadelphia, 1907-1911; F. F. Simpson, Pittsburg, 1907-1911; Frank Billings, Chicago, 1907-1910; George H. Monks, Boston, 1907-1910; L. S. McMurtry, Louisville, 1907-1909; Howard A. Kelly, Baltimore, 1907-1909; L. Emmett Holt, New York, 1907-1908.

Judicial Council: C. E. Cantrell, Texas; R. C. Cabot, Massachusetts; G. W. Guthrie, Pennsylvania; Thomas McDavitt, Minnesota; Charles J. Kipp, New Jersey.

